# Impact of the diagnosis definition on linkage detection

**DOI:** 10.1186/1471-2156-6-S1-S140

**Published:** 2005-12-30

**Authors:** Marie-Hélène Dizier, Emmanuelle Génin, Marie Claude Babron, Catherine Bourgain

**Affiliations:** 1 Hôpital Paul Brousse, Bâtiment Leriche, B.P. 1000, 94817 Villejuif Cedex, France

## Abstract

Previous genome scan linkage analyses of the disease Kofendrerd Personality Disorder (KPD) with microsatellites led to detect some regions on chromosomes 1, 3, 5, and 9 that were identical for the three populations AI, KA, and DA but with large differences in significance levels. These differences in results may be explained by the different diagnosis definitions depending on the presence/absence of 12 traits that were used in the 3 populations AI, KA, and DA. Heterogeneity of linkage was thus investigated here according to the absence/presence of each of the 12 traits in the 3 populations. For this purpose, two methods, the triangle test statistic and the predivided sample test were applied to search for genetic heterogeneity. Three regions with a strong heterogeneity of linkage were detected: the region on chromosome 1 according to the presence/absence of the traits a and b, the region on chromosome 3 for the trait b, and the region on chromosome 9 for the traits k and l. These 3 regions were the same as those detected by linkage analyses. No novel region was detected by the heterogeneity tests. Concerning chromosome 1, linkage analyses showed a much stronger evidence of linkage for traits a and b and for a combination of these traits than for KPD. Moreover, there was no indication of linkage to any of the other traits used to define the diagnosis of KPD. A genetic factor located on the chromosome 1 may have been detected here which would be involved specifically in traits a and b or in a combination of these traits.

## Background

Different diagnosis definitions depending on the presence/absence of 12 traits were used in 3 populations (AI, KA, and DA). These diagnosis definitions were not precisely known in each population. Linkage analyses of the Kofendrerd Personality Disorder (KPD) with microsatellites [1] were conducted using the model-free nonparametric linkage (NPL) statistic [2] in all 100 replicates for each population and led to detection of some regions on chromosomes 1, 3, 5, and 9, which were identical for the three populations but had large differences in significance levels. Such differences could be due either to the different diagnosis definitions or to different trait frequencies in the 3 populations. Our aim was to investigate heterogeneity of linkage with the disease according to the presence/absence of each trait used to define the diagnosis. In other terms, when linkage is detected between a region and the disease, we attempted to evaluate the impact of the presence/absence of each trait on the finding. For this purpose, we used two methods to detect genetic heterogeneity, the triangle test statistic (TTS) [3] and the predivided sample test (PST) [4]. These approaches may also permit detection of novel regions that were not detected by linkage analyses. We conducted a replication study to confirm regions detected in the first study. For regions, traits and populations with which heterogeneity of linkage were detected, linkage analyses were then conducted using the maximum likelihood score (MLS) method [5] in all subjects (affected or not), to evaluate the impact of the phenotype used in the analysis in the detection of linkage.

We had no knowledge of the "answers" at the time we did the analyses. Three of the 4 regions detected by previous linkage analyses of KPD were also detected by the heterogeneity tests: the regions on chromosomes 1, 3, and 9 according to the presence/absence of the traits a and b, the trait b, and the trait a, respectively. No novel regions were detected by these tests. For the region on chromosome 1, linkage analyses showed a much stronger evidence of linkage for the traits a and b and for a combination of these two traits than for KPD.

## Methods

### MLS method

The principle of sib-pair linkage analysis is to assess the sharing of marker alleles identical by descent (IBD) among affected sib-pairs and to conclude in favor of linkage if the observed IBD distribution differs from that expected under the null hypothesis of independent segregation of the disease and markers. This comparison can be made with the MLS statistic [5], based on a maximum likelihood method, which estimates the proportions of sib pairs with 2, 1, or 0 marker allele(s) (IBD z_2_, z_1 _and z_0_, respectively) given the observed marker genotypes. If a susceptibility gene is linked to the marker, the respective proportions z_2_, z_1_, and z_0 _are constrained within a triangle by: 2z_0 _≤ z_1 _≤ 0.5, referred to as the triangle constraints [6]. However, it has been shown that if the phenotype of each member of a sib pair is determined by different genetic models, i.e., corresponding to different genotype relative risks (relative penetrances), the triangle constraints may not be valid [3]. This situation can occur when the two sibs differ for a factor (e.g., severe vs. mild form of the disease) that modifies the genotype relative risks for the disease.

### TTS

The TTS [3] determines whether the triangle constraints in marker IBD distribution among affected sib-pairs are fulfilled. This test is defined as: TTS = log10(L(Z_u_)/L(Z_c_)) where L(Z_c_) is the likelihood of the IBD vector estimated with the triangle constraints, and L(Z_u_) is the likelihood without these constraints. The null hypothesis H_0 _is composite: absence of linkage or linkage with genetic homogeneity (i.e., relative penetrances are the same for the phenotypes of both members of the sib pair). Rejection of H_0 _means linkage with genetic heterogeneity within sib pairs, i.e., the sibs differ for a factor on which the relative penetrances depend. Derivation of TTS distribution and table of thresholds for various sizes are described in Dizier et al. [3,7]. As expected, power of the TTS is increased when it is applied only on sib pairs discordant for the phenotypes between which heterogeneity is investigated. This strategy was used here.

### PST

The PST tests whether IBD distributions in different groups of sib pairs are similar. Let us consider two sub-phenotypes, A and A'. Under the null hypothesis of genetic homogeneity (i.e., the same relative penetrances for the two sub-phenotypes), the Z_c _vectors estimated, with the triangle constraints, among pairs concordant for the phenotype A and the phenotype A' are expected to be equal. Therefore, the null hypothesis can be tested by PST = -2 [Ln(L(z_c1_) × L(z_c2_)/L(z_c_))]. When likelihoods are estimated in the different groups without constraints, the PST follows a χ^2 ^distribution with 2 df [4] (number of independent parameters × (number of categories -1)) where L(z_c1_), L(z_c2_) and L(z_c_) are the likelihoods of the parameter vector, Z_c_, estimated in respectively the concordant sib pairs for phenotype A, the concordant pairs for A' and the whole sample. Here, because the triangle constraints were applied to estimate the likelihoods and the parameter vector Z, the PST would not exactly follow a χ^2 ^with 2-df distribution. We showed by bootstrap analyses that the PST distribution was close to a χ^2 ^with 1-df distribution.

### Strategy of analysis

We conducted analyses to search for linkage heterogeneity using the information provided by the entire set of microsatellites covering the 10 chromosomes and 5 replicates of family samples (rep 1 to 5) for each of the 3 populations (AI, KA, and DA). These replicates were pooled for the analyses and led to a sample of 500 families for each population. This represents a reasonable size to apply tests of heterogeneity (i.e., sizes are sufficient in each category). The PST and TTS tests were applied on affected sib pairs to search for heterogeneity of linkage with KPD according to each trait consecutively. More precisely, the PST was applied on affected sib pairs concordant for the presence or the absence of the trait and the TTS on affected sib pairs discordant for the trait, i.e., one sib had the trait, the other did not. Only 7 traits (a, b, c, i, j, k, l) were considered because among affected subjects, some of the traits (e, f, h) were always present and some (d, g) were always confounded with c. To account for problems of multiple tests increasing type I error, we repeated genetic heterogeneity analyses for replications in a second set of 500 families (5 pooled replicates: rep 6 to 10). Only the populations, genomic regions, and traits that were found significant at a *p *≤ 0.005 in the first set of families were followed up in the second set. Results were considered replicated if the same genomic regions was significantly detected (*p *≤ 0.005) in the second sample for the same trait, the same population, and using the same method (TTS or PST). A genomic region was defined as including the marker with a maximum score in the first dataset and two adjacent markers at every side. For regions, traits, and populations with which genetic heterogeneity were detected, linkage analyses were conducted with the MLS method in the whole sample of subjects (i.e., affected or not). All tests (PST, TTS, MLS) were applied using all possible independent pairs in sibships (i.e., (n-1) pairs per sibships with n affected sibs). Analyses with these 3 tests were conducted using multipoint information on the microsatellites.

## Results and discussion

Three chromosomal regions led to a genetic heterogeneity (*p *≤ 0.005) simultaneously in the two sets of data for the same trait in the same population using the PST test (see Table 1, which presents these results). No regions fulfilled our criteria for replication with the TTS. A region on chromosome 1 at markers 23–25 was detected with the PST for the trait a in the 3 populations and for the trait b in AI (and in KA, but only in the second set of data), chromosome 3 at marker 42 for trait b with the PST in KA (and in AI only in the second set), and on chromosome 9 at markers 1–4 for the traits k and l with the PST in AI (and in KA, but only in the first set). Note that these 3 regions were also detected by linkage analysis of the disease KPD [1]. Thus, no novel region was detected by the heterogeneity tests. Note however that a region on chromosome 5 at markers 3–4, which was detected by linkage analysis of the disease KPD, was detected here for the trait a (*p *≤ 0.005) but not with the same methods and in the same populations for the two data sets: with TTS for the first set in DA and with PST in AI for the second set.

For these regions, detection of linkage with KPD depends strongly on the presence/absence of the traits for which genetic heterogeneity was shown. We retained the detected region on chromosome 1 at markers 23–25 for traits a and b for further analyses. Absence/presence of these traits modified strongly the IBD distribution among affected sib-pairs and thus the detection of linkage. The IBD distribution in the AI population was, in the first set, equal to (0.01, 0.5, 0.49) in 60 affected sib-pairs with the trait a vs. (0.20, 0.49, 0.31) in the 496 affected sib pairs without this trait. The IBD distributions were equal to (0.11, 0.48, 0.40) and (0.25, 0.5, 0.25), respectively, in the 358 and 148 affected sib pairs with and without trait b in the same population and set of data. In the absence of trait b, there was thus no longer IBD distortion from the IBD distribution expected under no linkage. Because traits a and b appeared to be important for the detection of linkage, we conducted separate and combined linkage analyses of these two traits in the whole sample of subjects (affected or not). We then considered the following phenotypes for linkage analyses: presence of trait a, presence of trait b, presence of both traits a and b, presence of trait a and/or of trait b and KPD. We conducted linkage analyses of these phenotypes in the first and second sets of data of the population AI (population in which the strongest heterogeneity was detected for traits a and b). Results are presented in Table 2 (only results obtained in the first set of data are presented because there were very similar to those obtained in the second set). Detection of linkage with the region on chromosome 1 at markers 23–25 was stronger for trait a and trait b than for KPD, although the number of sib pairs with KPD was greater than those for trait a and trait b and it was the strongest for trait b and trait a and/or b. The significance level of detection was quite equivalent for these two last traits. That could be due to the negligible difference of numbers of sib pair with trait b and with trait a and/or b. Also the less significant detection of linkage for trait a than for trait b could be due to a larger number of sib pairs with trait b (411) than with trait a (73). Note that linkage analysis of trait b among only affected subjects did not lead to a greater MLS than the one obtained in the whole sample (results not shown). That suggests that no other trait used for the diagnosis definition besides traits a and b had a large impact on the detection of linkage. Indeed, linkage analyses conducted separately for the each of traits other than a or b provided no indication of linkage with the chromosome 1 region (results not shown). Very similar conclusions were obtained in the population KA: although the MLS values are smaller for all the analyzed phenotypes than those obtained in the AI population, the much highest MLS is still obtained for the trait b.

### Comparison of our findings with the underlying model

In agreement with our results, the underlying model included a locus on chromosome 1 involved in both traits a and b, in affected and unaffected subjects. Concerning the linked region on chromosome 3, we found a significant heterogeneity according to the presence/absence of trait b in affected subjects. The underlying model indeed assumed a locus located in this region, involved in trait b and a combination of this trait with other traits, in affected subjects. For the linked region on chromosome 9, we found heterogeneity according to the presence/absence of traits k and l, which indeed depend on a locus located in this region according to the underlying model. The region in chromosome 5, which included a locus involved in trait a and in trait b combined with other traits, was not detected here with our criteria. However, heterogeneity of linkage was suggested with a *p*-value ≤ 0.005 according to the presence/absence of the trait a: with the TTS in DA in the first set, and with the PST in AI for the second set.

## Conclusion

Our analyses with the heterogeneity tests have permitted the detection of 3 of the 4 genetic factors, the 3 located on chromosomes 1, 3, and 9 and moreover to determine which of the 12 traits depend on these genetic factors. For example, we have detected here a genetic factor on chromosome 1 in the region of markers 23–25 that is not specifically involved in KPD disease, but which is involved in only some traits used to define the disease: traits a and b. Moreover, the much stronger detection of linkage between traits a and b with the chromosome 1 region than the one detected with KPD illustrates well the importance of the diagnosis definition in linkage analysis for the detection of linkage.

## Abbreviations

IBD: Identical by descent

KPD: Kofendrerd Personality Disorder

MLS: Maximum Likelihood score

NPL: Nonparametric linkage

PST: Predivided sample test

TTS: Triangle test statistic

## Authors' contributions

MHD conceived the design of the study, participated in the analysis and the interpretation of results, drafted the manuscript. EG carried out the heterogeneity test analyses and participated in the interpretation of results and the draft of the paper. MCB and CB participated in the analysis.

## Figures and Tables

**Table 1:** Regions detected for heterogeneity with either the PST or the TTS (*p *≤ 0.005) in the two sets of data

			First set		Second set	
				
Regions	Trait	Population	TTS	PST	TTS	PST
Chr 1 markers 23–25	a	AI	0	17.8^c ^(181.2 cM)^a^	0.3	10.8^d ^(173.6 cM)
		KA	0.02	10.3^d ^(173.6 cM)	0.3	10.8^d ^(173.6 cM)
		DA	0	11.3^d ^(189.5 cM)	0.1	16.3^c ^(173.6 cM)
	b^b^	AI	0.1	19.1^c ^(173.6 cM)	0.05	11.8^d ^(173.6 cM)
		KA	0.9	6.2^f ^(173.6 cM)	0.34	9.2^e ^(173.6 cM)
Chr 3 marker 42	b^b^	KA	0.1	11.5^d ^(287 cM)	0	14.3^d ^(287 cM)
		AI	0	4.5 (287 cM)	0	10^e ^(287 cM)
Chr 9 markers 1–4	k	AI	0.8	11.1^d ^(0.075 cM)	0.2	11.8^d ^(0.075 cM)
		KA	0.1	18.5^c ^(0 cM)	0.3	4.7 (0.23 cM)
	l	AI	0	9.4^e ^(0.075 cM)	0	7.9^e ^(0.135 cM)
		KA	0	19.1^c ^(0 cM)	0	6.6^f ^(0.075 cM)

^a ^distance between the first marker of the chromosome and the peak score

^b ^the trait b was present in all affected subjects in population DA and could not be tested here for heterogeneity.

^c ^*p *≤ 0.0001

^d ^*p *≤ 0.001

^e ^*p *≤ 0.005

^f ^*p *≤ 0.05

**Table 2:** Results of linkage analyses with the MLS method on chromosome 1 at markers 23–25 in the AI population

Phenotypes	Trait a	Trait b	Trait 'a and b'	Trait 'a and/or b'	KPD
Nb of sib-pairs^a^	73	411	65	421	622
Markers					
23	4.58^b,e^	10.75^c^	3.93^e^	10.91^c^	4.65^e^
24	6.75^c^	9.91^c^	5.40^d^	10.60^c^	3.86^f^
25	4.17^f^	6.38^c^	3.43^f^	6.84^c^	2.77^g^

^a ^with both sibs having the trait or the disease

^b ^MLS score

^c ^*p *≤ 10^-7^

^d ^*p *≤ 10^-6^

^e ^*p *≤ 10^-5^

^f ^*p *≤ 10^-4^

^g ^*p *≤ 10^-3^
